# Parameter Optimization of a Surface Mechanical Rolling Treatment Process to Improve the Surface Integrity and Fatigue Property of FV520B Steel by Machine Learning

**DOI:** 10.3390/ma17184505

**Published:** 2024-09-13

**Authors:** Yongxin Zhou, Zheng Xing, Qianduo Zhuang, Jiao Sun, Xingrong Chu

**Affiliations:** Associated Engineering Research Center of Mechanics and Mechatronic Equipment, Shandong University, Weihai 264209, China; 202020915@mail.sdu.edu.cn (Y.Z.); 202000800169@mail.sdu.edu.cn (Z.X.); 202017439@mail.sdu.edu.cn (Q.Z.); sunjiao1980@126.com (J.S.)

**Keywords:** support vector machine, surface integrity, surface mechanical rolling treatment, parameters optimization, active learning

## Abstract

Surface integrity is a critical factor that affects the fatigue resistance of materials. A surface mechanical rolling treatment (SMRT) process can effectively improve the surface integrity of the material, thus enhancing the fatigue property. In this paper, an analysis of variance (ANOVA) and signal-to-noise ratio (SNR) are performed by orthogonal experimental design with SMRT parameters as variables and surface integrity indicators as optimization objectives, and the support vector machine-active learning (SVM-AL) model is proposed based on machine learning theory. The entire model includes three rounds of AL processes. In each round of the AL process, the SMRT parameters with relative average deviation and high output values from cross-validation are selected for the additional experimental supplement. The results show that the prediction accuracy and generalization ability of the SVM-AL model are significantly improved compared to the support vector machine (SVM) model. A fatigue test was also carried out, and the fatigue property of the SMRT specimens predicted by the SVM-AL model is also higher than that of the other specimens.

## 1. Introduction

The centrifugal compressor is a key equipment in the petrochemical industry, and its reliability has an important impact on the safe transportation of flammable gas [1]. FV520B steel, as a commonly used material for centrifugal compressor impeller blades and other key load-bearing parts in the petrochemical industry, boasts advantages such as high strength, excellent corrosion resistance, and easy processing [2,3,4]. However, due to high load, high gas flow rate, and high-pressure ratio during use, the fatigue failure of the parts is inevitable. With the continuous improvement in the pressure ratio of centrifugal compressors, improving their service life is particularly important. According to the statistics, the fatigue failure of most parts occurs on the surface [5,6,7]. Therefore, without changing the existing materials, how to improve surface integrity is the key to prolonging the fatigue life of the parts. In recent years, to practice the concept of green manufacturing, the method to enhance the surface integrity and fatigue behavior of the parts through a surface mechanical strengthening treatment process has received considerable attention. Among them, the surface mechanical rolling treatment (SMRT) process can significantly improve the fatigue life of the parts [8,9,10]. This is because the SMRT process reduces the surface roughness of the sample, increases the degree of work hardening, introduces residual compressive stress, and improves the microstructure, thus hindering fatigue crack initiation and propagation [11,12,13].

There are several parameters in the SMRT process, including pressure (i.e., static force), feed rate, rolling speed, the number of passes, and tool diameter. Firstly, the pressure and the number of rolling passes are the most important parameters because they have the most direct effect on both the surface quality and plastic deformation of the material [14]. Secondly, the feed rate and rolling speed affect the surface integrity of the material by influencing the duration of action of the pressure per unit area of the material [15]. Thirdly, when the tool diameter is varied, it mainly affects the contact regions between the rolling tool and the workpiece, which in turn affects the pressure exerted on the material surface [16]. However, since the effect of pressure on the surface characteristics of the material is much greater than the tool diameter, the effect of the tool diameter on the surface characteristics was neglected in this study. The accurate acquisition of the optimized parameters generally requires a large number of experimental datasets, and fatigue testing is expensive and time-consuming, which all pose a great challenge for parameter optimization [17]. To reduce the experimental cost, many scholars have optimized the SMRT parameters based on the mapping relationship between surface integrity and fatigue life, using surface integrity as an indicator. For example, Attabi et al. [18] investigated the influence of SMRT parameters on the surface microhardness of 316L stainless steel by the response surface method. The results presented that the microhardness after SMRT process increased from 8.4% to 67.6% compared to the microhardness of the original specimen and the optimum parameters were obtained. The same approach was adopted by Reza et al. [19] to study the influence of an ultrasonic-assisted ball burnishing process on the surface integrity of AA6061-T6 aluminum alloy and to develop a prediction model for surface roughness and surface microhardness based on SMRT parameters. They found that the ultrasonic amplitude, the feed rate, and the static force were 8 µm, 1000 mm/min, and 38 N, respectively. The minimum surface roughness and the maximum surface microhardness were obtained simultaneously on the sample surface.

In addition, the influence of the SMRT process on fatigue properties has also been widely studied. For example, Liu et al. [20] investigated the fatigue properties of 17-4PH steel by the SMRT process. They found that the large residual compressive stress and high microhardness introduced by the SMRT process helped to improve the fatigue performance of 17-4PH steel. Among them, the highest fatigue life was obtained for the SMRT specimens after three passes. The fatigue life of the six-pass SMRT specimens decreased significantly due to higher surface roughness. Nalla et al. [21] improved the fatigue properties of Ti-6Al-4V titanium alloy at room temperature and 450 °C by the SMRT process. They concluded that the residual compressive stress induced by the SMRT process was the main factor in improving the fatigue life at room temperature. In addition, the residual stress was released at higher temperatures, but the SMRT process was still effective in improving the fatigue life of Ti-6Al-4V titanium alloy. Rodríguez et al. [22] used the SMRT process to improve the fatigue life of AISI 1038 steel by inducing surface work hardening and introducing surface residual stress. They believed that increasing the pressure and number of passes would result in a greater work hardening and higher fatigue life. In addition, they concluded that the surface-hardening effect and fatigue life are positively correlated. The effect of the SMRT process on the surface layer condition and fatigue life of steel welded joints was investigated by Dänekas et al. [23]. The results showed that the SMRT process can effectively improve the surface quality and increase the wear resistance and fatigue strength of steel welded joints. Oevermann et al. [24] studied the surface characteristics and fatigue life of the equiatomic high-entropy alloy CoCrFeMnNi through the SMRT process at low and room temperatures. The results showed that the residual compressive stress were greater in the SMRT specimen processed at room temperature. The SMRT specimen had a higher fatigue life compared to the untreated specimen. However, the fatigue life of the SMRT specimens processed at low and room temperatures was almost indistinguishable. In summary, improving surface integrity through the SMRT process to enhance the fatigue life of specimens is an effective method.

Recently, machine learning (ML) methods have achieved revolutionary success in several fields as the core technology of artificial intelligence strategies [25,26,27,28]. However, the relatively small sample dataset in material preparation compared to other industries limits the advantages of ML methods in materials science from being fully achieved. In this regard, machine learning methods have been developed to reach a high prediction accuracy by using limited experimental data [29,30,31]. For example, Yin et al. [32] also substantially improved the prediction accuracy of the ML model and reduced the amount of data for training through an orthogonal experimental design and data augmentation. Zhang et al. [33] and Liu et al. [34] improved the model’s ability to classify material components by incorporating material descriptors into the ML framework, thus improving the prediction accuracy of the model. In addition, there are some machine learning strategies used to predict the fatigue performance of metal materials. Luo et al. [35] statistically predicted the fatigue life of Inconel 718 alloy fabricated by selective laser melting (SLM) through the machine learning model. The results showed that the fatigue life was reduced by the increase in hole size, their number, and the proximity to the specimen surface. They also found that the location of the holes was the most critical factor affecting the fatigue life compared to the size and number of holes. The fatigue life of AlSi10Mg alloy was predicted using the same process by Salvati et al. [36]. They used a new prediction model, named physical information neural network (PINN). The prediction accuracy of the fatigue life was substantially improved compared to the classic Linear Elastic Fracture Mechanics (LEFM) model. Similarly, Mortazavi et al. [37] proposed a radial basis function artificial neural network (RBF-ANN) model to predict fatigue crack growth behavior. They also concluded that the new model has a better interpolation capability to predict the nonlinearity of short and long crack growth behavior compared to the LEFM model. He et al. [38] predicted the fatigue life of a material based on the S/N curve data of steel materials by means of the Random Forest (RF) and Artificial Neural Network (ANN) algorithms. Subsequently, based on the limited fatigue life data, they performed an inverse analysis via Bayesian optimization to determine the fatigue limit. The results showed that the machine learning model can accurately predict the fatigue life for most of the datasets. An inverse analysis was also able to estimate reasonable fatigue limits. Wei et al. [39] divided the model into two layers utilizing a transfer algorithm. The first layer was the prediction of static tensile properties by material composition and processing parameters, which had a dataset with a large sample size. The second layer related the tensile properties to the fatigue behavior using a small validated dataset of fatigue testing experiments and obtained a high prediction accuracy. The above attempts all demonstrate that ML is a potential method that can optimize the experimental parameters and the resultant fatigue property of metallic materials.

In the present work, we considered the mapping relationship between SMRT parameters and surface integrity, and used an experimental scheme combining orthogonal and complementary tests. First, the significance and trend of the surface integrity indicators for the SMRT parameters were revealed. Second, utilizing these experimental data, we propose a support vector machine of active learning (SVM-AL) model based on machine learning theory to predict the surface integrity indicators and validate the optimized SMRT parameters by surface integrity and fatigue testing experiments. We applied the modified machine learning model to the SMRT process to optimize of SMRT parameters for the surface integrity and fatigue performance of FV520B steel, which lays the theoretical and experimental foundation for extending the service life of centrifugal compressor impeller blades using the SMRT process.

## 2. Effect of SMRT Parameters on Surface Integrity

### 2.1. Material and SMRT Process

**Figure 1:**
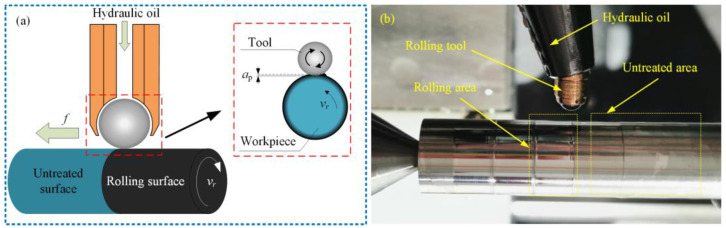
Schematic diagram of the SMRT process: (**a**) schematic diagram; (**b**) manufacturing process.

FV520B steel is a key manufacturing material for natural gas compressor blades, and its chemical compositions are demonstrated in Table 1. The original round bar samples with a diameter of 8 mm and a length of 60 mm were prepared by the finish turning (FT) process on a CNC lathe. The turning tool is a TNMG1600404-PM YBC251 carbide turning tool manufactured by ZCCCT (Zhuzhou, China). Subsequently, the samples were machined by the SMRT process on the same CNC lathe (Figure 1a,b). The HG6-9E270°-SL25-Z rolling tool was adopted through the production of Ecoroll (Celle, Germany). This study took the pressure P, feed rate *f*, rolling speed *v*_r_, and the number of passes *n* as independent variables, as well as residual stress, roughness and microhardness on the surface as optimization indexes. The experimental design used the quasi-level method to establish a mixed orthogonal table L_32_ (8^1^, 4^3^), which is shown in Table 2.

### 2.2. Surface Integrity Characterizations and Fatigue Test

In this work, the surface roughness was observed at a magnification of 20× using a VK-X1000 laser confocal microscope from Keyence (Osaka, Japan). Axial residual stress were measured using X-ray diffraction with an ST X-350A residual stress analyzer from Handan Aist Stress Technology (CrKα radiation, 156° diffraction angle, 60 s exposure time, 1 mm aperture diameter, Handan, China). The surface microhardness was measured using an MHV-100z microhardness tester from Shanghai Shangcai (Shanghai, China). The load was 0.98 N and the holding time was 10 s. The surface roughness, microhardness, and residual stress for each parameter were averaged over 3 measurements. It should be noted that the residual stress, roughness, and microhardness on the surface of FT process were 0.83 μm, 446 HV, and −289 MPa, respectively, before the SMRT process.

**Figure 2:**
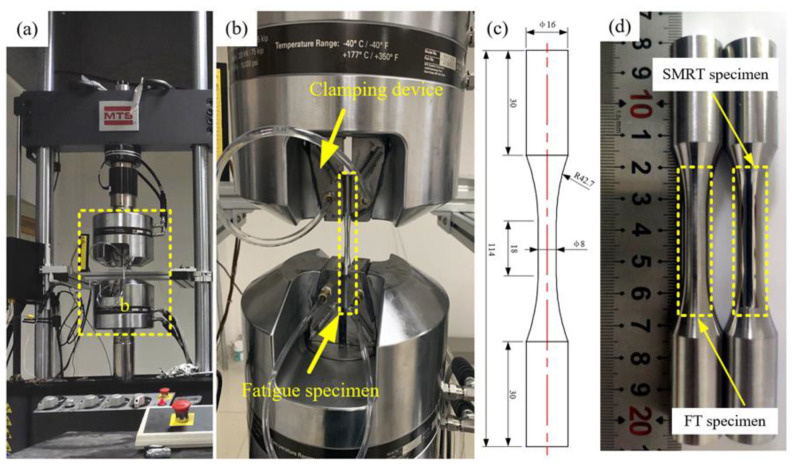
(**a**,**b**) Fatigue loading, (**c**) dimensions of the fatigue specimen (Unit: mm), and (**d**) FT and SMRT specimens.

Uniaxial tensile–compression fatigue tests were carried out at room temperature by using an MTS-809 electro-hydraulic testing system according to the GB/T 3075-2021 standard [40], as shown in Figure 2a,b. The stress ratio of the fatigue test was R = −1, the maximum fatigue stress was 900 MPa, and the frequency was 20 Hz. A sample with a test section diameter of 8 mm was used for the fatigue testing. The fatigue specimens and dimensions before and after the SMRT process are shown in Figure 2c,d. For each set of SMRT process parameters, three parallel specimens were tested to ensure the repeatability of the fatigue test.

### 2.3. Orthogonal Test Analysis

Statistical analysis was conducted using an analysis of variance (ANOVA) and signal-to-noise ratio (SNR) to assess the influencing trend of the selected variables on the evaluation indexes in the orthogonal experimental design presented in Table 3.

### 2.4. Analysis of Variance

The results of the analysis of ANOVA and the *F*-ratio test for roughness, microhardness, and residual stress on the surface are shown in Table 4, Table 5 and Table 6, respectively. According to the *F*-distribution table, the degree of freedom for pressure is 5; the degrees of freedom for the feed rate, rolling speed, and the number of rolling passes are 3; and the degree of freedom for error is 17. At a significance level of 0.05 (i.e., confidence level of 95%), *F*_0.05_(5,17) is 2.81 and *F*_0.05_(3,17) is 3.2. It can be seen from Table 4 that the *F*-ratio value (8.823) of *P* is greater than 2.81, the *F*-ratio values (69.644 and 6.646) of *f* and *n* are greater than 3.2, and the *F*-ratio value (1.805) of *v*_r_ is lower than 3.2. The P_F_-values (9.34 × 10^−10^, 0.005 and 0.0003) for *f*, n, and P are lower than 0.05. This indicates the surface roughness is significantly influenced by pressure, feed rate, and the number of passes. The parameters affecting the surface roughness in descending order of importance are: feed rate> pressure > the number of passes. Table 5 shows that the *F*-ratio value(1.396) of *P* is lower than 2.81, and the *F*-ratio values (2.041, 0.152 and 0.234) of *f*, *v*_r_, and *n* are also lower than 3.2. The conclusion was drawn that the SMRT parameters have no significant effect on the surface microhardness. Table 6 presents that the *F*-ratio value (7.94) of *P* is greater than 2.81, the *F*-ratio value (5.44) of *f* is also greater than 3.2, and the *F*-ratio values (0.45 and 0.72) of *v*_r_ and *n* are lower than 3.2. The P_F_-values (0.01 and 5.16 × 10^−5^) for *f* and P are lower than 0.05. It can be concluded that both the pressure and feed rate exert a notable influence on the surface residual stress, with pressure demonstrating a stronger impact compared to the feed rate.

### 2.5. Analysis of Signal-to-Noise Ratio

**Figure 3:**
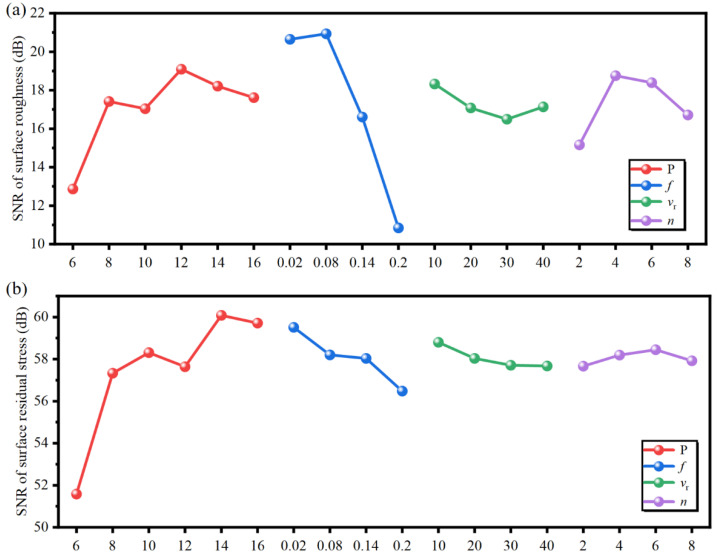
Effects of the SMRT parameters on SNR of (**a**) surface roughness and (**b**) surface residual stress.

In this paper, SMRT parameters had no significant effect on the surface microhardness. Therefore, only the roughness and residual stress on the surface were analyzed for the SNR. The SMRT process can effectively reduce the surface roughness, lower the stress concentration, delay the fatigue crack initiation, and thus improve the fatigue life. In addition, the SMRT process can also introduce residual compressive stress on the material surface, thus hindering fatigue crack propagation and improving the fatigue property. This indicates that a smaller surface roughness and higher surface residual compressive stress are more favorable to enhance the fatigue performance of FV520B steel. Therefore, Equations (1) and (2) can be used to calculate the SNR for the surface roughness and residual stress, respectively. A larger value of the calculated SNR represents a smaller surface roughness or a larger surface residual compressive stress.

(1) $$\eta_{i}=-10\mathrm{lg}\left(\frac{1}{k}\sum_{i=1}^{k}y_{i}^{2}\right)$$

(2) $$\eta_{i}=-10\mathrm{lg}\left(\frac{1}{k}\sum_{i=1}^{k}1/y_{i}^{2}\right)$$

where *k*, $y_{i}$, and $\eta_{i}$ are the number of tests, the *i*th experimental value, and the SNR, respectively. The influence of the SMRT parameters on the SNR of the surface roughness and residual stress is shown in Figure 3. It can be seen that the increase in pressure led to a rapid reduction in surface roughness (>6 MPa) and showed a small fluctuating change trend in the range of 8~16 MPa. The surface residual compressive stress showed an overall increasing trend with the increase in the pressure, but the magnitude of the increase becomes smaller and smaller. The surface roughness first remained almost constant with the increase in the feed rate, and then increased rapidly, which indicated that there may be a turning point in the feed rate that results in the rapid rise in the surface roughness. The surface residual compressive stress decreased with the increase in the feed rate, which may be related to the shorter duration of the SMRT process. As the number of passes increases, the surface roughness decreased and then increased, which indicated that the optimum number of passes may be between 4 and 6.

## 3. Prediction and Parameter Optimization

According to the results in Section 3, the SMRT parameters significantly influence both the surface residual stress and roughness. Therefore, based on machine learning theory, the measurements of surface residual stress and roughness were chosen to build the model for prediction and parameter optimization. Since, in a general support vector machine (SVM) regression model, there is only one output parameter (i.e., the prediction of the target value), in order to optimize both surface roughness and surface residual stress, the two output parameters were combined into a binding factor as the only optimization objective. In addition, in order to avoid a large difference in magnitude affecting the prediction accuracy of the model, logarithmic operations for residual stress and squaring operations for surface roughness were performe. The binding factor is expressed as:

(3) $$b_{f}=\frac{\ln\left|\sigma \right|}{e^{R_{a}}}$$

where $b_{f}$ is the binding factor, σ is the surface residual stress, and *R*_a_ is the surface roughness. When the binding factor is larger, it indicates that the surface roughness is smaller and the surface residual compressive stress is larger.

### 3.1. Support Vector Machine

Recently, great progress has been made in predicting material properties using the ML model [41,42,43]. However, the prediction accuracy and range of the prepared material properties have yet to be improved due to the high cost of experiments and the small sample size. Additionally, many other experiments with high experimental costs cannot achieve good results on some machine learning models due to the small amount of data. To this end, the SVM of the ML model based on statistical learning theory was developed. SVM regression can achieve a high prediction accuracy even with a small number of samples, and it can then use SVM classification to cross-validate the predicted results, thereby obtaining reliable optimization results [44].

SVM, a popular kernel learning method, utilizes the kernel function to map sample data into a higher-dimensional space for linear regression purposes [45,46]. Here is the detailed operational procedure.

#### 3.1.1. SVM Regression

Assume that the feature vector of the sample data is (*x_i_*,*y_i_*), where *x_i_* represents the input parameters and *y_i_* represents the output parameters. There exists a fitting parameter *f* (*x*), which is obtained by solving the function to predict the value of *y_i_* corresponding to *x*_i_ to accomplish the prediction task. The linear function of the SVM is shown in Equation (4).

(4) $$f\left(x\right)=\omega \cdot x+b$$

where ω and *b* are the weight variation and the fitted linear function coefficient, respectively. The regression problem of the fitted function can be viewed as a constrained optimization problem to find the optimal plane, as follows:

(5) $$\underset{\omega ,b,\xi ,\hat{\xi }}{\min}\left[\frac{1}{2}{\left\|\omega \right\|}^{2}+C\sum_{i=1}^{m}\left(\xi_{i}+{\hat{\xi }}_{i}\right)\right]$$

(6) $$s.t.\left\{\begin{array}{c} \left(\omega \cdot x_{i}+b\right)-y_{i}\leq \varepsilon +{\hat{\xi }}_{i}\\ y_{i}-\left(\omega \cdot x_{i}+b\right)\leq \varepsilon +\xi_{i}\\ \xi_{i}\geq 0,{\hat{\xi }}_{i}\geq 0,i=1,2,\ldots ,m \end{array}\right.$$

where ε is the insensitive loss function. ξ and $\hat{\xi }$ are slack variables. *C* is the penalty factor. To simplify the solution, Lagrange multipliers are utilized to convert the quadratic programing problem into a dual problem formulation. Firstly, the Lagrange function can be defined as follows:

(7) $$L\left(\omega ,b,\xi ,\hat{\xi },\alpha ,\hat{\alpha },\mu ,\hat{\mu }\right)=\frac{1}{2}{\left\|\omega \right\|}^{2}+C\sum_{i=1}^{m}\left(\xi_{i}+{\hat{\xi }}_{i}\right)-\sum_{i=1}^{m}\alpha_{i}\left[y_{i}-\left(\omega \cdot x_{i}+b\right)-\varepsilon -\xi_{i}\right]-\;\sum_{i=1}^{m}{\hat{\alpha }}_{i}\left[\left(\omega \cdot x_{i}+b\right)-y_{i}-\varepsilon -{\hat{\xi }}_{i}\right]-\sum_{i=1}^{m}\left(\mu_{i}\xi_{i}+{\hat{\mu }}_{i}{\hat{\xi }}_{i}\right)$$

where α and $\hat{\alpha }$ represent Lagrange multipliers. μ and $\hat{\mu }$ are introduced temporary variables. The partial derivation of $\omega ,b,\xi ,\hat{\xi }$ is presented in Equation (7) and the values of the partial derivative are set to zero.

(8) $$\left\{\begin{array}{c} \frac{\partial L}{\partial \omega }=0\Rightarrow \omega =\sum_{i=1}^{m}\left({\hat{\alpha }}_{i}-\alpha_{i}\right)x_{i}\\ \frac{\partial L}{\partial b}=0\Rightarrow 0=\sum_{i=1}^{m}\left({\hat{\alpha }}_{i}-\alpha_{i}\right)\;\;\;\;\;\\ \frac{\partial L}{\partial \xi }=0\Rightarrow C=\alpha_{i}+\mu_{i}\;\;\;\;\;\;\;\;\;\;\;\;\;\;\;\\ \frac{\partial L}{\partial \hat{\xi }}=0\Rightarrow C={\hat{\alpha }}_{i}+{\hat{\mu }}_{i}\;\;\;\;\;\;\;\;\;\;\;\;\;\;\; \end{array}\right.$$

The solved $\omega ,b,\xi ,\hat{\xi }$ are brought into the Lagrangian function and the Karush–Kuhn–Tucker (KKT) conditions are applied to the Lagrangian function. This optimal regression problem is transformed into a dual problem, as follow:

(9) $$\underset{\alpha ,\hat{\alpha }}{\max}\left[\sum_{i=1}^{m}y_{i}\left({\hat{\alpha }}_{i}-\alpha_{i}\right)-\varepsilon \left({\hat{\alpha }}_{i}+\alpha_{i}\right)-\frac{1}{2}\sum_{i=1}^{m}\sum_{j=1}^{m}\left({\hat{\alpha }}_{i}-\alpha_{i}\right)\left({\hat{\alpha }}_{j}-\alpha_{j}\right)K\left(x,x_{i}\right)\right]$$

(10) $$s.t.\left\{\begin{array}{c} \sum_{j=1}^{m}\left({\hat{\alpha }}_{i}-\alpha_{j}\right)=0\\ 0\leq \alpha_{i},{\hat{\alpha }}_{i}\leq C,\;\;i=1,2\ldots ,m \end{array}\right.$$

where $K\left(x,x_{i}\right)$ is the kernel function. Through solving the aforementioned equation, the optimal regression hyperplane of SVM regression be formulated as:

(11) $$f\left(x\right)=\sum_{i=1}^{m}\left({\hat{\alpha }}_{i}-\alpha_{i}\right)K\left(x,x_{i}\right)+b$$

The common kernel functions are shown below:

(1) Linear function

(12) $$K\left(x,x_{i}\right)=x\cdot x_{i}+c$$

(2) Radial basis function

(13) $$K\left(x,x_{i}\right)=exp\left(-\frac{\|x-x_{i}{\|}^{2}}{2\sigma^{2}}\right)$$

(3) Poly function

(14) $$K\left(x,x_{i}\right)={\left(a\left(x\cdot x_{i}\right)+c\right)}^{d}$$

**Figure 4:**
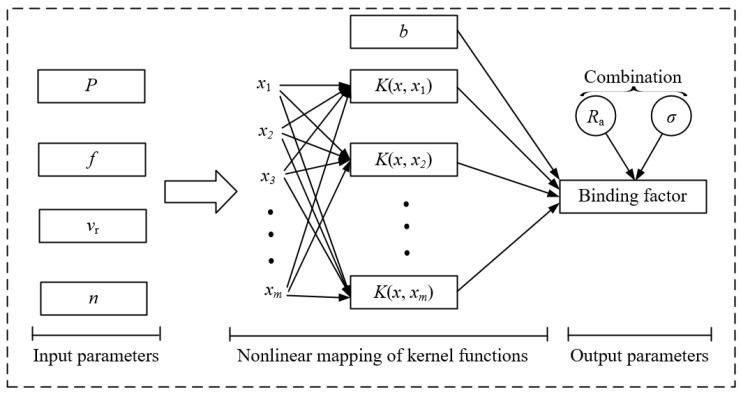
Schematic of the operations of SVM regression.

(4) Sigmoid function

(15) $$K\left(x,x_{i}\right)=\tanh\left(a\left(x\cdot x_{i}\right)+c\right)\;$$

where *a*, *c*, *d*, σ are the kernel function parameters. A schematic of the operation of the SVM regression model can be obtained based on the operations of Equations (4)–(13), as shown in Figure 4.

The coefficient of determination *R*^2^ and relative average deviation (*RAD*) are used to assess the predicted accuracy and generalizability of the SVM model, as shown in Equations (16) and (17).

(16) $$R^{2}=\frac{{\left(n\sum_{i=1}^{n}f\left(x_{i}\right)y_{i}-\sum_{i=1}^{n}f\left(x_{i}\right)\sum_{i=1}^{n}y_{i}\right)}^{2}}{\left(n\sum_{i=1}^{n}f{\left(x_{i}\right)}^{2}-{\left(\sum_{i=1}^{n}f\left(x_{i}\right)\right)}^{2}\right)\left(n\sum_{i=1}^{n}y_{i}^{2}-{\left(\sum_{i=1}^{n}y_{i}\right)}^{2}\right)}$$

(17) $$RAD=\frac{1}{n}\sum_{i=1}^{n}\frac{1}{y_{i}}\left(ny_{i}-\sum_{i=1}^{n}f\left(x_{i}\right)\right)$$

where *n*, $y_{i}$, and $f\left(x_{i}\right)$ are the sample size, the actual value, and the predicted value of validation experiments, respectively.

#### 3.1.2. SVM Classification

It is assumed that there exists a hyperplane that can separate the data points as accurately as possible. This hyperplane can be represented as:

(18) ω·x+b=0

The problem of finding the optimal classification hyperplane is represented by a constrained optimization scheme, as follow:

(19) $$\underset{\omega ,b}{\min}\left(\frac{1}{2}{\left\|\omega \right\|}^{2}\right)$$

(20) $$s.t.\;y_{i}\left(\omega \cdot x_{i}+b\right)\geq 1,i=1,2,\ldots ,m$$

To simplify the problem, Lagrange multipliers can be introduced. The Lagrange function can be defined as:

(21) $$L\left(\omega ,b,\alpha \right)=\frac{1}{2}{\left\|\omega \right\|}^{2}+\overset{m}{\underset{i=1}{\sum }}\alpha_{i}\left[1-y_{i}\left(\omega \cdot x_{i}+b\right)\right]$$

where α is Lagrange multiplier. The Lagrangian function is transformed into a dual problem using a solution similar to SVM regression.

(22) $$\underset{\alpha }{\max}\left[\sum_{i=1}^{m}\alpha_{i}-\frac{1}{2}\sum_{i=1}^{m}\sum_{j=1}^{m}\alpha_{i}\alpha_{j}y_{i}y_{j}K\left(x,x_{i}\right)\right]$$

(23) $$s.t.\;\sum_{i=1}^{m}\alpha_{i}y_{i}=0\;\left(\alpha_{i}\geq 0,\;i=1,2\ldots ,m\right)$$

where $K\left(x,x_{i}\right)$ is the kernel function. Through solving the aforementioned equation, the optimal classification hyperplane of SVM be formulated as:

(24) $$f\left(x\right)=sign\left[\sum_{i=1}^{m}\alpha_{i}y_{i}K\left(x,x_{i}\right)+b\right]$$

where sign is the sign function. If $\sum_{i=1}^{m}\alpha_{i}y_{i}K\left(x,x_{i}\right)+b>0$, $f\left(x\right)=1$. If $\sum_{i=1}^{m}\alpha_{i}y_{i}K\left(x,x_{i}\right)+b<0$, $f\left(x\right)=-1$.

### 3.2. SVM-AL Model

**Figure 5:**
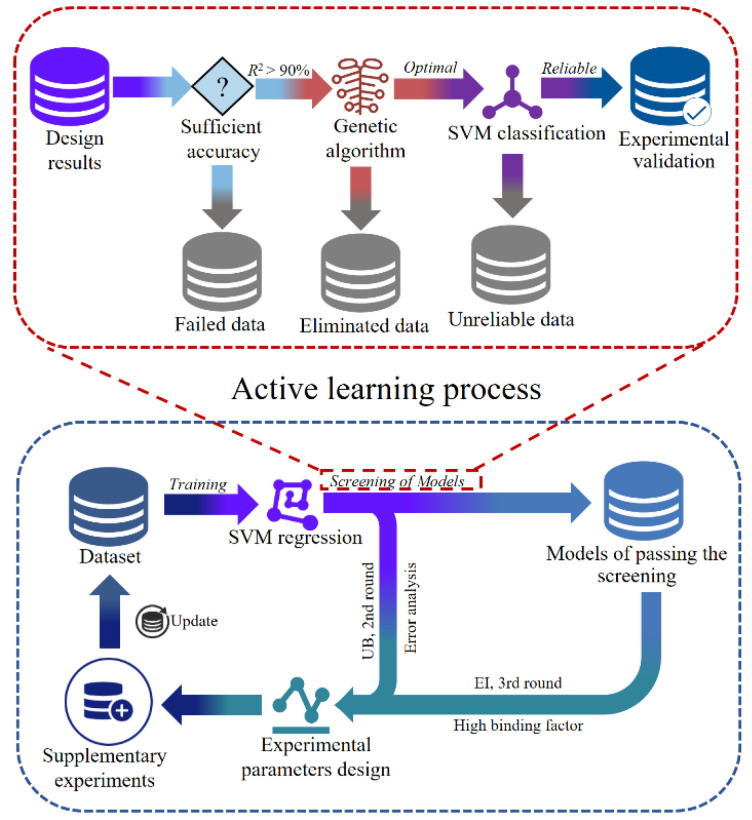
Flow diagram for the prediction and validation of the SVM-AL model.

SVM model is considered as an effective and reliable method to deal with small sample datasets due to its efficient model complexity control and powerful generalizability. However, the SVM model is very sensitive to missing data. To avoid model performance degradation due to missing data in small sample datasets, an active learning (AL) strategy was added to the SVM model [47], as shown in Figure 5.

The operation procedure of SVM-AL model was to first import the original dataset into the SVM regression model for the first round of training. Secondly, data points in the region around those with large prediction errors were selected for experimentation using uncertainty-based (UB) sampling [48], and the resulting experimental data were added to the original dataset for the second round of regression model training. Thirdly, data points in the region around those with high binding factors were selected for experimentation through expected improvement (EI) sampling [49], and the resulting experimental data were added to the total dataset for the third round of regression model training. Finally, the reliable regression prediction model was obtained. In addition, the predictive models trained in each round were screened by Genetic Algorithm (GA) and SVM classification models to obtain the optimized parameters for each round.

In this paper, the original dataset was composed of 32 sets of experimental data generated by the orthogonal experiments [32]. Compared to the SVM model, the SVM-AL model is supplemented with more critical data points to ensure that a higher prediction accuracy and generalizability are obtained. Notably, the AL process consists of two main operations: screening and updating of the model.

#### 3.2.1. Screening of SVM-AL Model

To enhance the assessment of the generalizability of the SVM model, this screening work used the “multiple hold-out method” [44], in which 80% of the dataset was allocated for training and the remaining 20% for testing, to construct 2000 different SVM models, and evaluate the generalization ability and prediction accuracy of these models based on three different criteria. The models with *R*^2^ > 0.9 were considered to pass the first evaluation. The second criterion was to use a genetic algorithm to iterate over the testing set that passed the first evaluation to obtain better parameters and larger binding factors. If the predicted value of the optimal solution of the genetic algorithm iteration was better than the optimal value of the initial population, the model was considered to have passed the second evaluation. The third criterion was cross-validation by the SVM classification model. If the output of the SVM classification model was low, the data were eliminated, while if the output of the SVM classification model was high, the data were considered to have passed the third evaluation and the passed data were recorded.

#### 3.2.2. Updating of SVM-AL Model

The addition of supplemental data to the SVM-AL model is achieved through UB sampling and EI sampling. UB sampling was used in the second round of supplemental data. The core of UB sampling is a measure of model uncertainty that leads to finding the data point with the smallest confidence level (equivalent to the largest error) to identify and select it, as shown in Equation (25). Subsequently, points in the region adjacent to these data points are then sampled and supplemented to the original dataset, thus improving the prediction accuracy of the model. The second round of supplementary data is shown in Table 7.

EI sampling was used in the third round of supplemental data. EI sampling needs to clarify the specific goal of sampling, so as to have directional and purposive sampling to improve the efficiency and effectiveness of active learning, as shown in Equation (26). In this study, data points with high binding factors were first screened in the prediction model. Then, points in the region near these data points were sampled and added to the total dataset to improve the prediction accuracy of the model specifically in the region with high binding factors, thereby facilitating the discovery of more reliable optimized parameters. The supplemental data for the third round is shown in Table 8.

(25) $$UB=argmax_{x}\left(1-P_{\theta }\left(y|x\right)\right)$$

(26) $$\mathrm{EI}=argmax_{x}\left(f_{\theta }\left(x_{i}\right)\right)$$

where θ denotes the set of parameters of a trained machine learning model. $P_{\theta }\left(y|x\right)$ and $f\left(x_{i}\right)$ are the selection functions for UB sampling and EI sampling of supplementary samples, respectively.

### 3.3. Results and Discussion

#### 3.3.1. The Optimization of Kernel Functions on the SVM-AL Model

**Figure 6:**
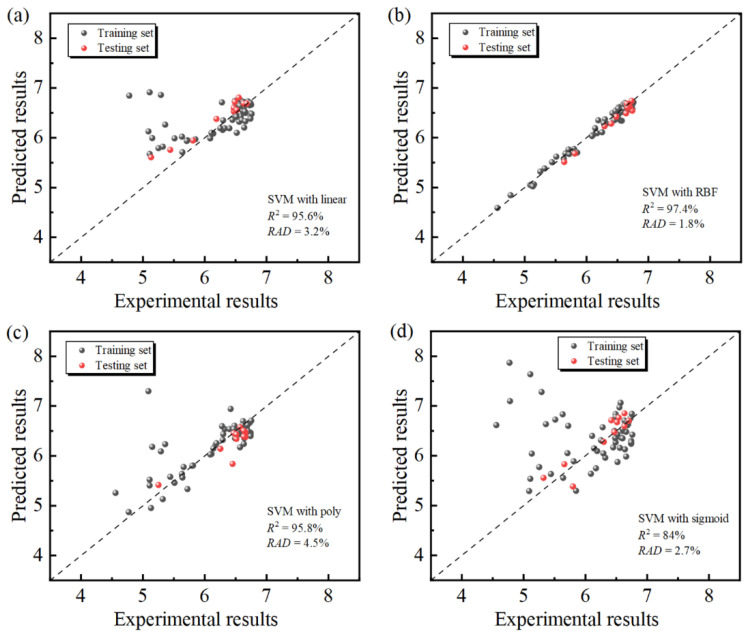
Experimental vs. predicted binding factors with different kernel functions of both the training and testing datasets, *RAD* and *R*^2^: (**a**) linear kernel; (**b**) RBF kernel; (**c**) poly kernel; and (**d**) sigmoid kernel.

The effect of different kernel functions (radial basis function (RBF) kernel, linear kernel, poly kernel, and sigmoid kernel) on the prediction accuracy and generalizability of the SVM-AL model were compared based on the total dataset, using the identical training and testing sets. The predicted and experimental values of the training and testing sets, as well as the *R*^2^ and *RAD* of SVM-AL models with different kernels, are shown in Figure 6. According to the comprehensive comparison, the *R*^2^ of all kernels except the sigmoid kernel is above 90%, and the *R*^2^ of the RBF kernel is the largest, reaching 97.4%. However, the further observation of the distribution of the predicted and experimental values revealed that the *RAD* of the poly and linear kernels are larger, and more points are randomly scattered in the region, which is poorly fitted. In contrast, the data points of RBF kernel are mostly around the diagonal. In addition, the training set of RBF kernel is also more concentrated, and its distribution is the same as that of the testing set. This may be because, in the sample preparation for third iteration of the SVM-AL model, most of the supplemented data belong to the region of high output values to enhance the fit of the model in the range of high output values, so the amount of data in the region of low output values are smaller, resulting in a slightly larger prediction error in this region. The RBF kernel had a generally better property for the SVM-AL model, which is consistent with previous studies on the prediction of material properties for small samples [42,50].

#### 3.3.2. The Optimization of Partitioning Parameters for the SVM-AL Model

**Figure 7:**
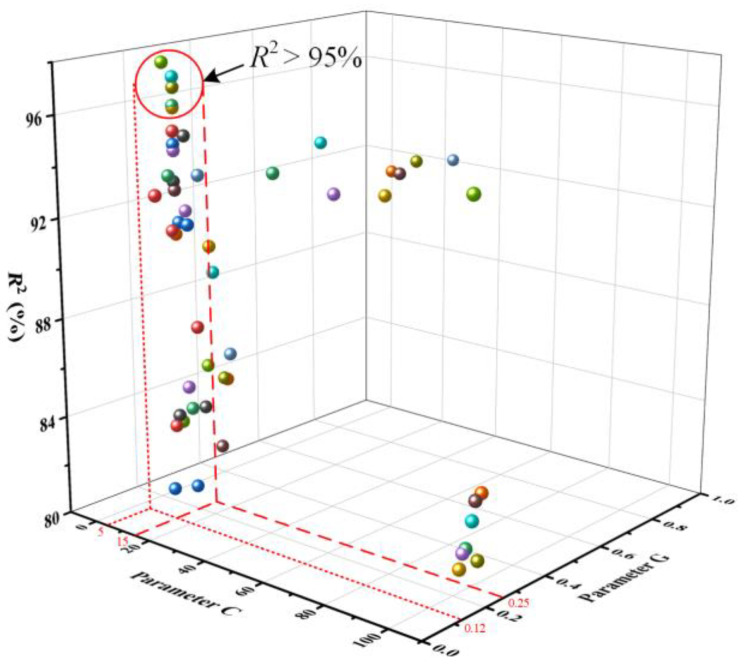
*R*^2^ distribution of SVM-AL models with different parameters C and G.

Parameters C and G are the most important parameters in the RBF kernel that significantly affect the prediction accuracy and generalizability of the SVM model [51]. When the values of the parameters C and G are too large or too small, it can lead to the poor prediction accuracy and generalizability of the SVM model. Therefore, to improve the accuracy of the model, the values of parameters C and G are optimized. The parameter C ranged from 1 to 100, and G ranged from 0.1 to 1. The corresponding *R*^2^ is shown in Figure 7. It can be observed that as the parameters C and G vary, the *R*^2^ fluctuates within the range of 80.6% to 97.4%. When C = 5~15 and G = 0.12~0.25, there exist multiple SVM-AL models with *R*^2^ above 95%. Also, when C = 10 and G = 0.15, *R*^2^ is the largest, indicating that the model has the highest prediction accuracy.

#### 3.3.3. Screening by SVM Classification Model

**Figure 8:**
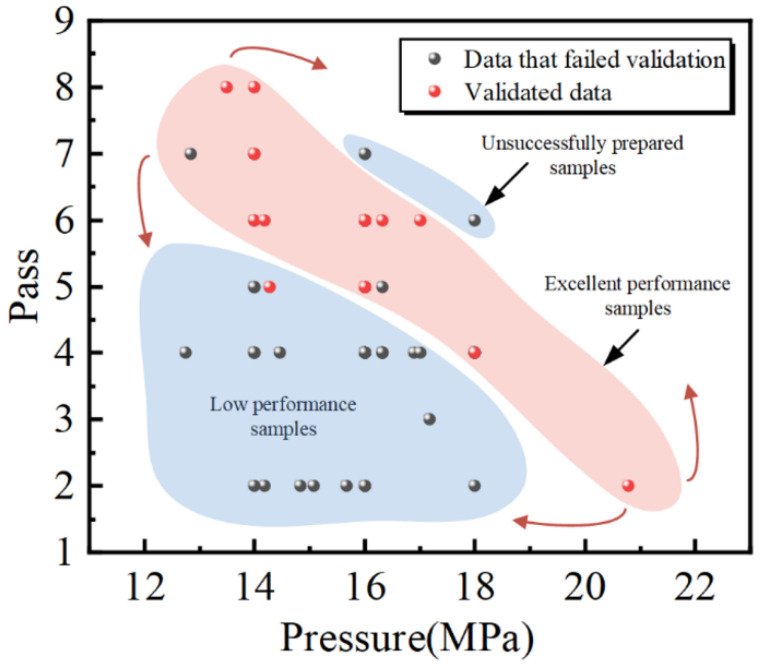
The distribution of SMRT parameters screened by the SVM classification model in the coordinate system of pressure and the number of passes.

To test the screening effect of SVM classification models, the design results that passed and failed the SVM classification model screening were analyzed. The design results included 112 solutions. Among them, 19 solutions passed the SVM classification model screening, while 93 solutions did not pass the SVM classification model screening. A comprehensive analysis of the data screened by the SVM classification model showed that the results can be presented by the scatter plot of the pressure and the number of passes, as shown in Figure 8. Most of the data that do not pass the model screening are located in the blue area at the bottom left of the figure, where the material produced by the parameters corresponding to this area does not have the desired properties. In the blue area at the top right of the figure, some models also fail the model screening, and the material produced by the parameters in this area will be broken due to excessive pressure or rolling passes, resulting in the unsuccessful prepared samples. Most of the data points that pass the model screening are concentrated in the red area, which represents samples of materials with high binding factors screened on the basis of guaranteed normal preparation. The results show that most design results with poor reliability can be effectively filtered out through the SVM classification model in the AL process, which improves the accuracy of prediction.

From the overall region, a balance between pressure and rolling pass is needed to prepare materials with a high binding factor. For a given pressure, the number of rolling passes needs to be chosen within a small range to produce a high-property material. Too many rolling passes can lead to the surface breakage of the material, while too few rolling passes can lead to a low binding factor of the material. When the number of rolling passes is certain, the change in pressure will also lead to the same change pattern. This phenomenon is similar to the study of the rolling process of 17-4PH steel [51]. Based on this law, the SMRT parameters of different materials can be optimized, and then the experimental parameters with low reliability can be precisely excluded, which improves the training accuracy and experimental efficiency.

#### 3.3.4. The Effect of AL Process on SVM Model

**Figure 9:**
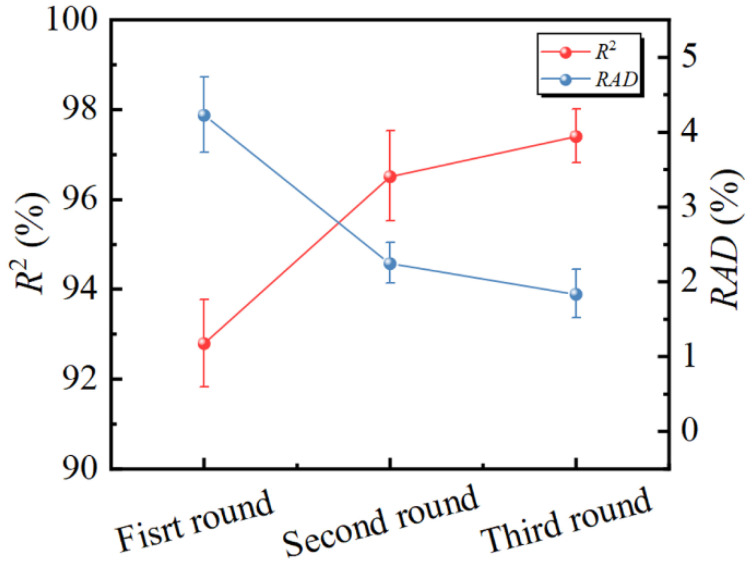
*R*^2^ and *RAD* for three rounds of AL process.

It is important to highlight that the AL process is essential for developing a dependable ML model. Therefore, a more detailed analysis of the AL process was conducted. *R*^2^ and *RAD* for each round of the AL process are shown in Figure 9. It can be observed that the *R*^2^ of the binding factor improves from 92.8% to 97.4%, and the *RAD* decreases from 4.2% to 1.8% as the number of rounds of the AL process increases. Compared to the first round of AL process, the *R*^2^ for the second round of AL process increases by 3.72% and the *RAD* decreases by 2.4%. The *R*^2^ for the third round of AL process increases by 0.89% and the *RAD* decreases by 0.004 in comparison to the second round of AL process. This indicates that the AL process can significantly improve the accuracy and stability of the model in fewer iterations in comparison to the traditional method of randomly supplementing data [47].

#### 3.3.5. Experimental Validation of the SVM-AL Model

**Figure 10:**
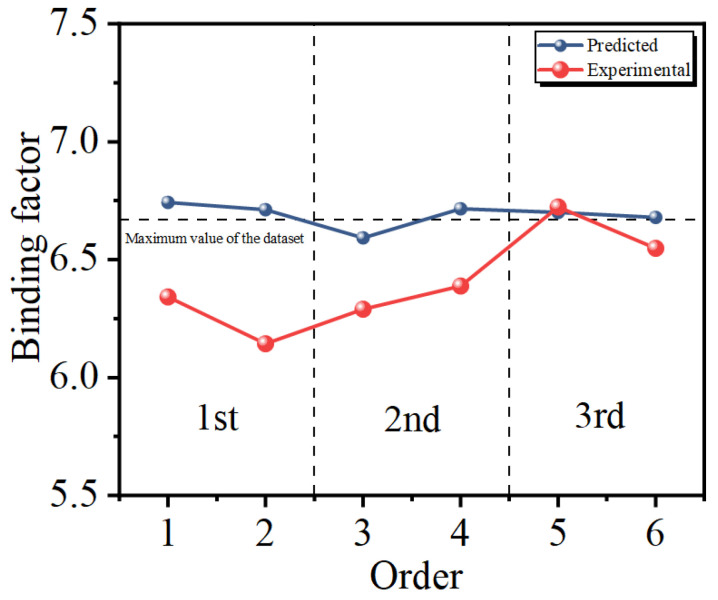
Predicted and experimental binding factors of the optimized parameters.

The optimized SMRT parameters predicted by the SVM-AL model for each iteration and their corresponding predicted and measured values are shown in Table 9 and Table 10, respectively. It can be noticed from Figure 10 that the binding factors of the optimized SMRT parameters are at the same level as the maximum values in the dataset. In addition, as the number of iterations increases, the measured binding factor increases. The difference between experimental and the predicted binding factors becomes smaller.

**Figure 11:**
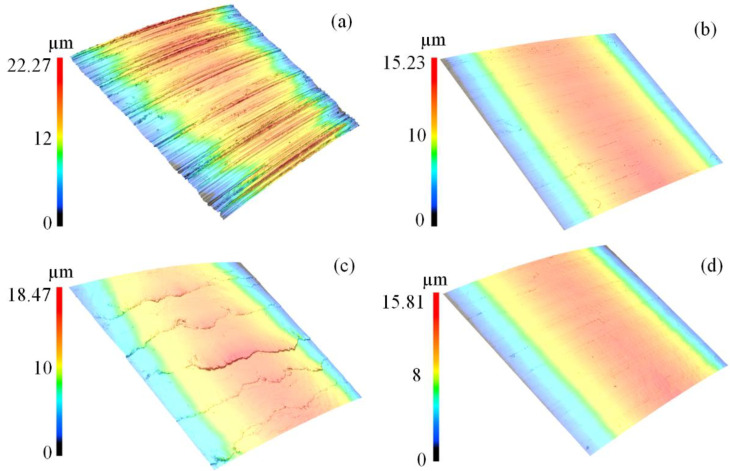
Surface topography of (**a**) turning specimen, (**b**) 24th in Table 3 (minimum surface roughness in the dataset), (**c**) 13rd specimen in Table 7 (maximum surface roughness in the dataset), and (**d**) 5th specimen of optimized parameters.

The surface morphology of FT and SMRT specimens (24th specimen in Table 3 (minimum surface roughness, 0.052 μm), 13rd specimen in Table 7 (maximum surface roughness, 0.446 μm)), and 5th specimen of optimized parameters (0.051 μm) are shown in Figure 11. It can be noticed that the surface roughness can be significantly decreased by the SMRT process, but the excessive treatment by the SMRT process caused the surface roughness to increase. In addition, although the 24th specimen in Table 3 has a lower surface roughness, the surface morphology with optimized parameters has less surface defects than that of the 24th specimen in Table 3.

**Figure 12:**
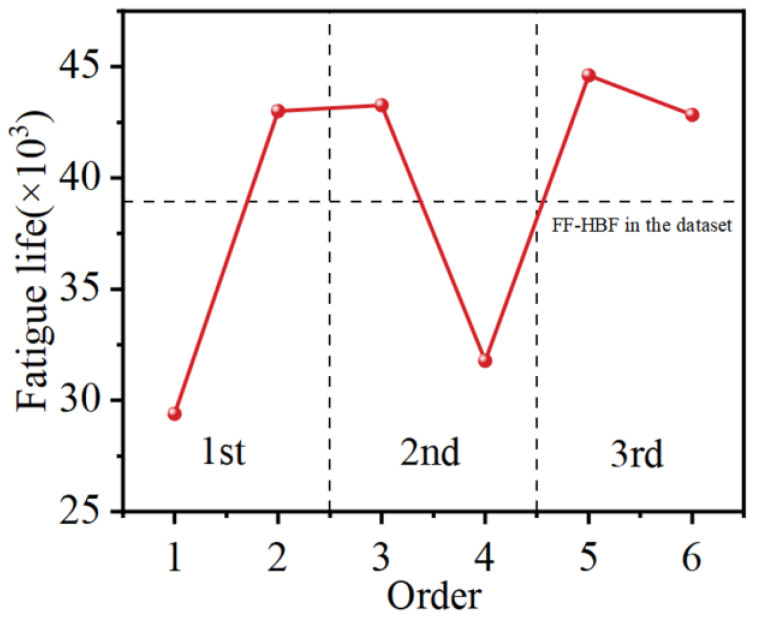
Fatigue life of optimized SMRT parameters of each iteration of the SVM-AL model.

The fatigue behavior of specimens machined with the optimized parameters is obtained in Figure 12. It can be noticed that the fatigue life of the highest binding factor (FF-HBF) in the dataset is also at the same level as the fatigue life of the optimized SMRT parameters. Furthermore, the fatigue life of the material prepared with the parameters predicted by the first iteration of the model is not stable. The discrepancy in fatigue life between the two parameters is more than 10,000 cycles. After the AL process, the discrepancy between the two predicted parameters of the second iteration of the model decreased slowly, while the discrepancy between the two predicted parameters of the third iteration of the model is only 1773 cycles. This indicates that the AL process can sufficiently enhance the prediction accuracy and generalizability of the SVM-AL model, but achieving this goal requires generally several iterations of AL process. In this study, it is possible to rapidly promote the accuracy of the model with a smaller number of iterations due to the combination of UB and EI sampling schemes.

## 4. Conclusions

In this paper, the influences of SMRT parameters on the surface integrity (surface residual stress, microhardness, and roughness) and fatigue life are studied through an orthogonal experimental design and a machine learning prediction model. The specific conclusions are as follows:

(1) According to the orthogonal experimental analysis, the feed rate, pressure, and number of passes notably influence the surface roughness, the pressure, and feed rate have a greater effect on the surface residual stress, and the SMRT parameters have no significant effect on the surface microhardness. In addition, a larger pressure (8~16 MPa), a smaller feed (0.02~0.08 mm/r), and a suitable number of rolling passes (4~6) can decrease significantly the surface roughness. The larger the pressure, the smaller the feed rate, and the greater the surface residual compressive stress that can be obtained.

(2) The SVM-AL model based on the RBF kernel function has the best evaluation indexes (*R*^2^ = 97.4%, *RAD* = 1.8%). Compared to the SVM model, the evaluation indexes *R*^2^ and *RAD* of the SVM-AL prediction model increased by 4.6% and decreased by 57.1%, respectively. The prediction accuracy of the SVM-AL model is the best when C = 10 and G = 0.15, and the SVM classification model can effectively screen out invalid predictive parameters.

(3) As the number of iterations increases, the predicted and experimental results are more and more in agreement. Experimental validation through fatigue testing reveals that the fatigue property of the SMRT specimens with the predicted optimized parameters was in general agreement with that of the highest binding factor in the dataset.

(4) In this study, the ML model was used to optimize the SMRT parameters to obtain excellent surface integrity, and thus improve the fatigue life of the material. In the future, we will explore the effect of the SMRT parameters on fatigue life in more depth and develop fatigue life prediction models for more accurate and efficient predictions.

## Figures and Tables

**Table 1:** Chemical compositions of FV520B steel (wt. %).

C	Si	Mn	P	S	Ni	Cr	Cu	Nb	Mo	Fe
≤0.07	≤0.07	≤1.0	≤0.03	≤0.03	5.0~6.0	13.2~14.5	1.3~1.8	0.25~0.45	1.3~1.8	Bal.

**Table 2:** Parameter levels of the SMRT process.

	Parameters			
	Pressure P (MPa)	Feed Rate *f* *(mm/r)*	Rolling Speed *v*_r_ (m/min)	The Number of Passes *n*
Level 1	16	0.2	40	8
Level 2	14	0.14	30	6
Level 3	12	0.08	20	4
Level 4	10	0.02	10	2
Level 5	8	-	-	-
Level 6	6	-	-	-
Level 7	16 (quasi-level)	-	-	-
Level 8	14 (quasi-level)	-	-	-

**Table 3:** Orthogonal experimental results.

Order	Pressure (MPa)	Feed Rate (mm/r)	Rolling Speed (m/min)	The Number of Passes	Surface Roughness *R*_a_ (μm)	Surface Microhardness (HV)	Surface Residual Stress (MPa)
1	16	0.08	20	4	0.089	494.50	−986
2	16	0.02	10	2	0.109	488.47	−1148
3	10	0.02	20	4	0.08	492.31	−942
4	16	0.2	20	2	0.257	500.01	−923
5	16	0.08	40	6	0.072	494.40	−892
6	8	0.08	30	2	0.111	502.52	−704
7	10	0.14	40	8	0.138	501.09	−842
8	12	0.14	40	2	0.159	501.58	−841
9	16	0.14	30	6	0.115	496.85	−1261
10	8	0.2	10	6	0.242	504.31	−723
11	8	0.14	20	8	0.172	502.67	−682
12	14	0.02	30	2	0.087	490.57	−1280.5
13	14	0.14	30	4	0.119	498.77	−1137
14	8	0.02	40	4	0.07	494.95	−845
15	14	0.02	10	8	0.094	487.54	−1324
16	12	0.02	20	6	0.075	489.96	−955
17	6	0.2	10	4	0.28	507.75	−458
18	10	0.2	30	6	0.284	503.05	−674
19	16	0.14	10	4	0.114	496.63	−973.5
20	12	0.2	30	4	0.242	501.96	−458
21	6	0.08	30	8	0.209	503.03	−336
22	14	0.2	40	2	0.288	501.84	−598
23	16	0.2	40	8	0.285	498.34	−742
24	12	0.08	10	8	0.052	495.56	−917
25	14	0.2	20	8	0.283	499.05	−934.5
26	16	0.02	30	8	0.126	487.15	−905
27	6	0.02	40	6	0.136	497.03	−477
28	14	0.08	20	6	0.063	495.11	−1117
29	10	0.08	10	2	0.123	499.51	−858
30	14	0.08	40	4	0.066	496.31	−1044
31	6	0.14	20	2	0.334	507.69	−282
32	14	0.14	10	6	0.114	497.25	−861

**Table 4:** ANOVA of surface roughness.

Control Factors	Sum of Squares	dof	Mean Squares	F	P_F_
P	0.034	5	0.0068	8.823	0.0003
*f*	0.161	3	0.0537	69.644	9.34 × 10^−10^
*v*_r_	0.004	3	0.0014	1.805	0.184
*n*	0.014	3	0.0047	6.146	0.005
Error	0.012	17	0.0008		
Total	0.225	31			

**Table 5:** ANOVA of surface microhardness.

Control Factors	Sum of Squares	dof	Mean Squares	F	P_F_
P	733.831	5	146.766	1.396	0.275
*f*	643.695	3	214.565	2.041	0.146
*v*_r_	48.007	3	16.002	0.152	0.927
*n*	73.769	3	24.590	0.234	0.871
Error	1786.806	17	105.106		
Total	3286.108	31			

**Table 6:** ANOVA of surface residual stress.

Control Factors	Sum of Squares	dof	Mean Squares	F	P_F_
P	1,329,751	5	265,950	11.684	5.16 × 10^−5^
*f*	353,664	3	117,888	5.179	0.01
*v*_r_	60,516	3	20,172	0.886	0.468
*n*	8388	3	2796	0.123	0.945
Error	386,936	17	22,761		
Total	2,139,257	31			

**Table 7:** Second round of AL process supplementary data.

Order	Pressure (MPa)	Feed Rate (mm/r)	Rolling Speed (m/min)	The Number of Passes	Surface Roughness *R*_a_ (μm)	Surface Residual Stress (MPa)	Binding Factor
1	17	0.020	10	5	0.109	−1222	6.374
2	18	0.020	18	4	0.097	−1244	6.467
3	14	0.020	19	8	0.085	−1220	6.528
4	20	0.038	28	2	0.127	−1318	6.327
5	16	0.11	30	6	0.084	−1012	6.362
6	16	0.17	30	6	0.175	−955	5.760
7	15	0.2	40	8	0.229	−746	5.261
8	17	0.2	40	8	0.279	−836	5.090
9	16	0.01	10	4	0.095	−1224	6.466
10	18	0.08	20	4	0.085	−1229	6.534
11	16	0.086	21	7	0.072	−1162	6.568
12	16.33	0.023	18	4	0.096	−1149	6.402
13	20	0.001	14	2	0.446	−571	4.063
14	16	0.031	9.74	6	0.097	−1107	6.361
15	18	0.012	25	2	0.126	−1137	6.203
16	16	0.013	8.31	5	0.099	−1255	6.462
17	22	0.02	10	3	0.295	−591	4.751
18	24	0.02	10	2	0.320	−528	4.552
19	26	0.02	10	2	0.439	−546	4.064
20	28	0.05	15	2	0.427	−478	4.025

**Table 8:** Third round of AL process supplementary data.

Order	Pressure (MPa)	Feed Rate (mm/r)	Rolling Speed (m/min)	The Number of Passes	Surface Roughness *R*_a_ (μm)	Surface Residual Stress (MPa)	Binding Factor
1	16	0.08	10	4	0.077	−998	6.394
2	14	0.02	10	4	0.074	−884	6.301
3	16	0.02	10	4	0.077	−1114	6.496
4	14	0.08	20	4	0.111	−862	6.049
5	18	0.02	10	4	0.093	−978	6.274
6	20	0.02	10	4	0.096	−1057	6.326
7	12	0.02	10	4	0.059	−829	6.335
8	16	0.02	10	1	0.105	−1015	6.233
9	14	0.02	30	6	0.082	−1077	6.432
10	16	0.02	10	3	0.103	−1148	6.356
11	18	0.08	10	4	0.084	−1123	6.458
12	16	0.02	10	5	0.075	−1103	6.500
13	16	0.05	20	5	0.115	−1110	6.250
14	16	0.02	10	7	0.084	−1081	6.423
15	8	0.05	20	4	0.172	−394	5.032
16	16	0.02	10	9	0.097	−927	6.200
17	12	0.05	40	4	0.118	−791	5.931
18	16	0.08	10	4	0.061	−853	6.349
19	18	0.02	20	2	0.114	−1401	6.464
20	16	0.14	10	4	0.1	−840	6.093

**Table 9:** Predicted optimized parameters for each round of AL process.

Order	Iteration	Pressure (MPa)	Feed Rate (mm/r)	Rolling Speed (m/min)	Rolling Pass
1	1st	16	0.05	21.7	4
2	1st	14	0.13	25.8	4
3	2nd	16	0.05	14	5
4	2nd	18	0.02	18	4
5	3rd	17	0.02	8.5	5
6	3rd	14.8	0.02	10	8

**Table 10:** Predicted and experimental surface roughness, surface residual stress, and binding factor of optimized parameters.

Order	Surface Roughness (μm)		Surface Residual Stress (MPa)		Binding Factor	
	Predicted	Measured	Predicted	Measured	Predicted	Measured
1	0.067	0.097	−1357	−1083	6.743	6.343
2	0.068	0.134	−1318	−1125	6.712	6.143
3	0.073	0.099	−1205	−1037	6.592	6.290
4	0.062	0.086	−1272	−1053	6.716	6.388
5	0.048	0.051	−1134	−1184	6.701	6.724
6	0.065	0.072	−1248	−1134	6.680	6.547

## Data Availability

The original contributions presented in the study are included in the article, and further inquiries can be directed to the corresponding author.
